# Neuroblastoma of the Urinary Bladder in an Infant

**DOI:** 10.1055/s-0039-1692192

**Published:** 2019-06-17

**Authors:** Ahmed Mohamed, Quentin Campbell-Hewson, Hany O. S. Gabra

**Affiliations:** 1 Department of Paediatric Surgery, University Hospitals of Leicester NHS Trust, Leicester Royal Infirmary Infirmary Square Leicester, Leicester, United Kingdom of Great Britain and Northern Ireland; 2 Department of Paediatric Surgery, Royal Victoria Infirmary, Newcastle, United Kingdom

**Keywords:** neuroblastoma, pediatric oncology, urinary bladder, pediatric tumors

## Abstract

As it originates from neural crest cells, Neuroblastoma (NBL) can arise anywhere along the sympathetic chain. However, its occurrence in the urinary bladder (UB) is extremely rare. We present a case of an incidentally diagnosed pelvic NBL arising from the dome of the UB in a 7-month-old infant. The mass was treated with surgical excision only after being classified as a very low risk group according to the International Neuroblastoma Risk Group staging system. The patient was disease free after 5 years of follow-up. Although rare, we suggest that NBL should be considered in the differential diagnosis of UB masses in children and investigated accordingly.

## Introduction

Neuroblastoma (NBL) is the commonest solid tumor of infancy and the second most common extracranial malignant tumor of childhood. 1 Most commonly occurring in the abdomen in 65% of cases, NBL can also develop in the paraspinal sympathetic ganglia at other sites including the chest (20%), neck (5%), and pelvis (5%) 2_._ Forty percent of symptomatic patients are younger than 1 year at the time of diagnosis and with a slight male predominance (1.2:1). 1 NBL affects 1 in 7,000 children and is thought to be responsible for 15% of oncology-related mortality in childhood. 3 We report an unusual case of NBL arising from the dome of the urinary bladder (UB) of a 7-month-old infant. To the best of our knowledge, this is only the ninth reported case of a neuroblastic tumor originating primarily from the UB.

## Case Report

Our patient is a 7-month-old previously healthy boy, who was referred to our tertiary center with an incidental finding of a pelvic mass on ultrasound (US) that was performed as part of the investigative pathway for the febrile urosepsis he was being managed for.

**Fig. 1:**
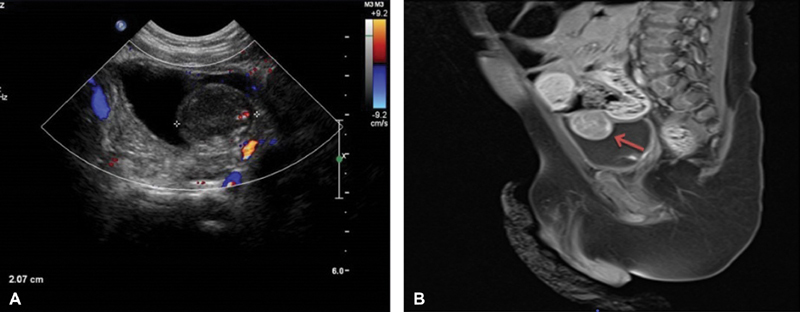
( **A** ) Incidentally discovered urinary bladder mass. ( **B** ) Magnetic resonance imaging (MRI) showing solid mass arising from the urinary bladder fundus ( *arrow* ).

Clinical examination and laboratory work-up were essentially unremarkable. US showed a well-circumscribed, solid, polypoid mass arising from the UB fundus, measuring 2 cm in diameter, with hypervascularity on color Doppler ( Fig. 1A ). Magnetic resonance imaging (MRI) showed a (20 × 18 × 17 mm) well-defined, homogenous, solid mass arising from the middle/left side of the dome of UB ( Fig. 1B ). A cystoscopic biopsy was attempted but proved to be difficult as the mass was completely submucosal and not clearly visible.

**Fig. 2:**
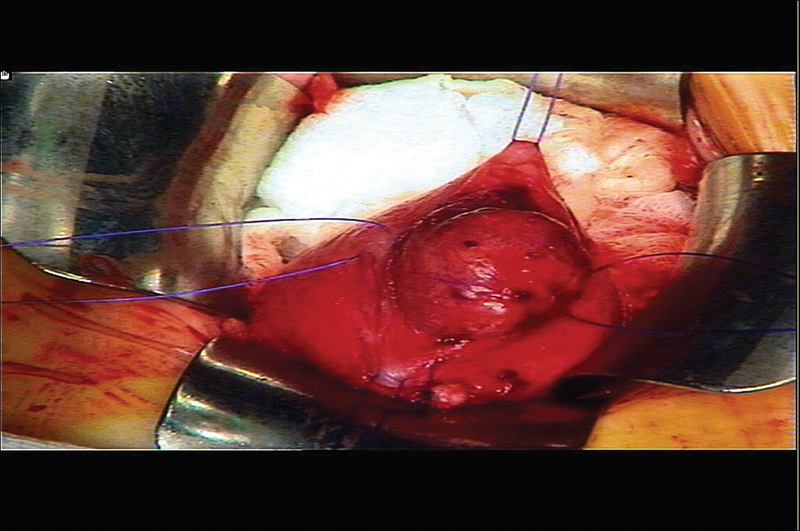
Intraoperative view of the urinary bladder mass.

We proceeded to excisional biopsy through an extended suprapubic incision. The dome of the UB was opened, and the tumor felt elastic, firm, and homogenous ( Fig. 2 ). The mass was excised completely with grossly negative margins, and the recovery period was uneventful. Histopathology was consistent with a completely excised, poorly differentiated NBL with low mitosis–karyorrhexis index and favorable histology, as per Shimada's classification. Multiplex ligation dependent probe amplification analysis showed no evidence of proto-oncogene N-myc (MYCN) amplification or any segmental chromosomal abnormalities.

Further assessment was made for metastatic disease, including bone scan, bone marrow biopsy, and MIBG (metaiodobenzylguanidine) scan, which were all negative. Urine creatinine, HMMA (4-hydroxy-3-methoxy mandelic acid)/creatinine, and homovanillic acid (HVA)/creatinine ratios were all within normal limits.

Due to favorable prognostic factors, our patient was classified as a very low risk group according to the International Neuroblastoma Risk Group staging system (INRGSS) and was treated by surgical excision only. Annual follow-up with clinical examination and two MRI scans performed after 1 and 3 years postoperatively revealed no evidence of recurrence or residual tumor. The patient remained asymptomatic throughout and was discharged from follow-up after 5 years.

## Discussion

NBL is the most common extracranial solid tumor of childhood. 4 NBL of the UB, however, is an almost unknown entity. As it originates from neural crest cells, NBL can occur anywhere along the sympathetic chain and has a median age of presentation of 18 to 23 months. 1 5 Most primary tumors occur in the abdomen in 65% of cases, commonly from the adrenal glands, and 15 to 20% occur in the thorax, whereas pelvic presentation is seen in approximately 5% of cases. 2 6 Rare sites of primary NBL include the orbit, lung, and genitourinary tract. 7 Tumors arising from the retroperitoneal or pelvic space are more frequently diagnosed on routine mass screening such as the Japanese program, which started in 1984, in which urine samples from infants aged 6 months were tested for HVA and vanilmandelic (VMA) acid by high-performance liquid chromatography. 8 9

Other than cases diagnosed by screening, bladder NBL can present with microscopic hematuria and/or the presence of a palpable mass on physical examination. 10 11 Our patient was asymptomatic, most likely due to the early incidental diagnosis. Literature review suggests eight other similar reported cases to date. Six patients were under 15 months of age, 12 whereas the other two patients were 3 and 5 years of age, respectively. 13 14 Interestingly, in six out of eight reported cases, the masses originated from the dome of the UB. 12 13 14 The two other reported sites of pathology were the lateral and the anterior walls of the UB. 12 13 14 All pathological subtypes of the NBL spectrum were described with four patients exhibiting poorly differentiating NBL, one case of stroma-poor differentiating NBL, one ganglioneuroblastoma (GNBL), a single ganglioneuroma (GN) and one case was not specified. 12 13 14

Table 1 summarizes the histological classification, MYCN amplification, chromosomal abnormalities, and treatment given for the described cases. All cases had a good outcome with no reported mortality. 12 13 14

**Table 1:** Characteristics of eight reported cases of neuroblastoma of the urinary bladder

Case	Age	Histology	Shimada's classification	MYCN	Chromosomal deletions	Ploidy	Surgery	Chemotherapy
1	3 mo	Poorly differentiated NBL	Favorable histology	Amp	Yes	Diploidy	Partial cystectomy	Adjuvant after recurrence
2	4 mo	Poorly differentiated NBL	Favorable histology	NA	–	Aneuploidy	Partial cystectomy	Adjuvant
3	4 mo	GNBL	–	–	–	–	Partial cystectomy	No
4	7 mo	Poorly differentiated NBL	Favorable histology	NA	–	–	Partial cystectomy	No
5	8 mo	Poorly differentiated NBL	Favorable histology	NA	No	–	Partial cystectomy	No
6	15 mo	NBL (not specified)	Favorable histology	NA	–	–	Partial cystectomy	Neoadjuvant
7	3 y	Differentiating NBL	Favorable histology	NA	–	–	Partial cystectomy	No
8	5 y	GN	–	–	–	–	Transurethral resection	No

Abbreviations: Amp, amplified; GN, ganglioneuroma; GNBL, ganglioneuroblastoma; MYCN, proto-oncogene N-myc; NA, not Amplified; NBL, neuroblastoma.

The preoperative staging system in NBL is the INRGSS of 2009, which has simplified the staging into L1, L2, M, and MS. 15 By a combination of postoperative staging and other prognostic factors including age, Shimada grade, histological features, and genetics, patients are grouped into four prognostic risk groups: very low, low, moderate, and high. 15 16 Postsurgery, NBL is classically staged from 1 to 4 according to the 1993 revised International Neuroblastoma Staging System. 17

NBL is known to exhibit the highest rate of spontaneous regression among malignant tumors. 18 Ghazali 19 first reported a series of pelvic NBL that underwent spontaneous regression and maturation. Subsequent studies adopting a watchful waiting approach in patients with NBL detected by screening or during infancy report high rates of regression. 20 21 Therefore, although active intervention is the mainstay of treatment, a wait-and-see approach avoiding surgical procedures and chemotherapy in localized NBL diagnosed in early infancy has been suggested.

Treatment strategies for NBL include surgery, chemotherapy, and/or radiation. Stem cell transplant and immunotherapy are reserved for advanced or recurrent disease. Low and intermediate risk groups have a 95% survival rate with the use of surgery and/or chemotherapy. High-risk groups with combination therapy have a survival between 40 and 50%. 5

Our patient was stage L1 by INRGSS and because of the favorable prognostic factors was put in the very low-risk group, which is amenable to treatment by surgical excision solely. Annual clinical examination and two postoperative MRI scans revealed no evidence of recurrent or residual tumor. We discharged the patient after 5 years of follow-up.

## Conclusion

Although rare, NBL should be considered in the differential diagnosis of pediatric urinary bladder masses, especially when arising from the dome.

## Notes

New Insights and the Importance for the Pediatric Surgeon Neuroblastoma should be considered in the differential diagnosis of urinary bladder masses occurring in the pediatric age group, especially when arising from the dome.
